# In vitro maturation in the presence of Leukemia Inhibitory Factor modulates gene and miRNA expression in bovine oocytes and embryos

**DOI:** 10.1038/s41598-020-74961-6

**Published:** 2020-10-20

**Authors:** Meritxell Vendrell-Flotats, Tania García-Martínez, Iris Martínez-Rodero, Manel López-Béjar, Jonathan LaMarre, Marc Yeste, Teresa Mogas

**Affiliations:** 1 Department of Animal Medicine and Surgery, Autonomous University of Barcelona, 08193 Cerdanyola del Vallès, Spain; 2 Department of Animal Health and Anatomy, Autonomous University of Barcelona, 08193 Cerdanyola del Vallès, Spain; 3 Department of Biomedical Sciences, Ontario Veterinary College, University of Guelph, Guelph, ON N1G 2W1 Canada; 4 Department of Biology, Institute of Food and Agricultural Technology, University of Girona, 17004 Girona, Spain

**Keywords:** Animal breeding, Gene expression, Gene regulation

## Abstract

Members of the interleukin-6 (IL-6) family of cytokines are important for reproductive function that are mediated through changes in gene and miRNA expression. Herein, we characterized the expression of miR-21, miR-155, miR-34c and miR-146a in bovine oocytes and cumulus cells during in vitro maturation (IVM) with leukemia inhibitory factor (LIF), IL-6 and IL-11 or unsupplemented controls. LIF-exposed COCs showed higher expression of miR-21 and miR-155 in oocytes, whereas miR-146a expression was increased in oocytes matured with IL-6 and IL-11. In cumulus cells, miR-155 expression was elevated by all treatments while only LIF increased miR-21 expression. Based on these results, we next examined how LIF exposure during IVM affected oocyte competence, through IVF and the expression of specific genes in GV- and MII-oocytes, in 2- and 8-cell embryos, and in Day 8-blastocysts. LIF supplementation did not affect cleavage rate, blastocyst yield or several other developmental parameters, but did increase hatching rate. LIF suppressed *DPPA3, ZAR1* and *NPM2* expression in 2 cell- and/or 8-cell embryos. LIF increased the expression of *KAT2A* and *HSPA1A* in MII-oocytes, and that of *HDAC1*, *KAT2A* and *HSP90AA1* and the *BAX:BCL2L1* ratio in 2-cell embryos. In contrast, *HDAC1, KAT2A* and *HSP90AA1* expression and *BAX:BCL2L1* ratio was lower in 8-cell embryos derived from LIF oocytes. IVM with LIF also increased the expression of *DNMT3A*, *HSPA1A* and *HSP90AA1* in blastocysts. In conclusion, supplementation with LIF during IVM was consistently associated with changes in the relative abundance of transcripts in mature bovine oocytes and in specific embryo developmental stages.

## Introduction

Oocyte differentiation occurs through complex processes beginning at the embryonic life and extending until the ovulation of a metaphase II stage (MII) oocyte. The correct series of events, occurring during this period of time, as well as the continuous dialog between the germ and somatic cells within the follicle, determine oocyte quality and pregnancy rates^1^. Understanding the complex pathways that regulate oocyte growth and maturation is not only important for basic science, but also represents the first step to enhance embryo and pregnancy outcomes in reproductive biotechnology. In this context, regulation of gene expression represents one of the most important cellular processes determining oocyte quality. Oocyte largely depends on maternal transcripts stored in the oocyte and others that are delivered from the surrounding somatic cells; these transcripts are used for meiosis resumption and during the first stages of development before embryo genome activation^2,3^. Therefore, a close-regulated communication between the oocyte and surrounding cumulus cells is necessary at various stages, including oocyte growth, maturation and fertilization^4^. Nonetheless, how exactly cumulus cells participate in the fertilizing ability and embryo developmental competence of the oocyte remains unknown. Different studies have focused on mRNAs, evidencing the importance of the oocyte transcriptome in determining oocyte quality^5^; such changes may be related to the microRNA (miRNA)-mediated regulation of transcript levels^6,7^.

MicroRNAs (miRNAs) are noncoding RNAs approximately 22 nucleotides in length that inhibit target gene expression through degrading or destabilizing cognate mRNA targets^6^. miRNAs influence various cellular processes, including cell proliferation, differentiation and apoptosis^8^. Recent studies have shown that miRNA levels change during oocyte maturation and ovarian follicular development implying a regulatory role^9,10^. miRNAs including miR-21^11,12^, miR-155^9,13^ and miR-34c are expressed or consistently change their expression patterns from the GV oocyte to the 2-cell embryo stage^14^. miR-146a is a potent regulator of the immune response and inflammation^15^. In addition, miRNA expression has been characterized in cumulus cells^16^, and the differentially-expressed miRNAs between corona radiata and outer cumulus cells from bovine oocytes supports a role for these miRNAs in signaling processes between oocytes and cumulus cells^7,16^.

Cytokines were first characterized as key participants in inflammation but have more recently been recognized to play vital roles in many processes such as follicular development and ovulation. Three members of the IL-6 family of cytokines, interleukin-6 (IL-6), interleukin-11 (IL-11) and LIF, are particularly important in the reproductive context (reviewed by Heinrich et al.^17^). IL-6 was originally identified as a B-cell differentiation factor that is produced by a number of cell types, and has been reported to play important roles in inflammation, immunity and hematopoiesis^18^. IL-6 has been detected in human follicular fluid and granulosa cells^19–21^, and IL-6 transcripts are present throughout development from zygote to the blastocyst stage^22^ and appear to be involved in cumulus expansion^23^. IL-11 is primarily an anti-inflammatory cytokine that was initially described as a growth factor in hematopoiesis, driving megakaryocyte differentiation and synergizing with other factors (reviewed by Du and Williams^24^). IL-11 is produced in preovulatory follicles by theca cells in response to LH and stimulates progesterone production and follicle development^25^. LIF was first described as a protein factor that induces differentiation in M1 murine myeloid leukemic cells^26^. It has been detected in human follicular fluid and granulosa cells^27^, and *LIF*-transcripts have also been found in mature bovine oocytes and early embryos^28^. It plays a crucial role during follicular development and ovulation, promoting primordial to primary follicle transition in rats^29^ and to coordinate follicular growth and ovulation in mice^30^. LIF has also been reported to promote oocyte maturation in mice^31^, cattle^32^, pigs^33^ and goats^34^ when added to maturation medium. In addition, murine and bovine oocytes matured with LIF lead to higher blastocyst rates^31,32^.

In addition to improved blastocyst yield, oocyte maturation in environments with high LIF or other IL-6 family members would be expected to induce significant changes in gene expression. In particular, alterations in the levels of maternal-effect genes (MEG) induced by LIF could be expected given their important roles in embryo gene activation (EGA)^35^. Furthermore, since LIF suppresses apoptosis either through the induction of miR-21 via STAT3^36^ or through BCL2L1 inhibiting caspase 3 via PI(3)-kinase/AKT^37^, it may drive a similar effect in the maturing oocyte. The combined effects of MEG and miRNA alterations may then participate in improved developmental outcomes. In this study, we propose that the identification of specific differentially expressed mRNAs and miRNAs during in vitro maturation, together with the assessment of transcript levels of genes during subsequent bovine embryo development could help us understand the effect of changing the maturation milieu on oocyte quality. To this end, we first evaluated the levels of key miRNAs known to be expressed in oocytes and cumulus cells (miR-21, miR-155, miR-34c and miR-146a) after in vitro maturation with IL-6 family cytokines. We then investigated the effect of supplementing in vitro maturation medium with LIF in embryo development after fertilization and inner cell mass (ICM) and trophectoderm (TE) cell distribution and apoptosis. Transcript abundances of maternal-effect genes [zygote arrest 1 (*ZAR1*), nucleoplasmin 2 (*NPM2*), developmental pluripotency associated 3 (*DPPA3*)], and genes related to epigenetics (*DNMT3A*, *KAT2A* and *HDAC1*), heat stress (*HSPA1A*, *HSP90AA1*) and apoptosis (*BAX*, *BCL2L1*) were determined in bovine MII oocytes and day 8 blastocysts.

## Results

### Effect of IL-6, IL-11 and LIF on miRNA expression in oocytes and cumulus cells

**Figure 1:**
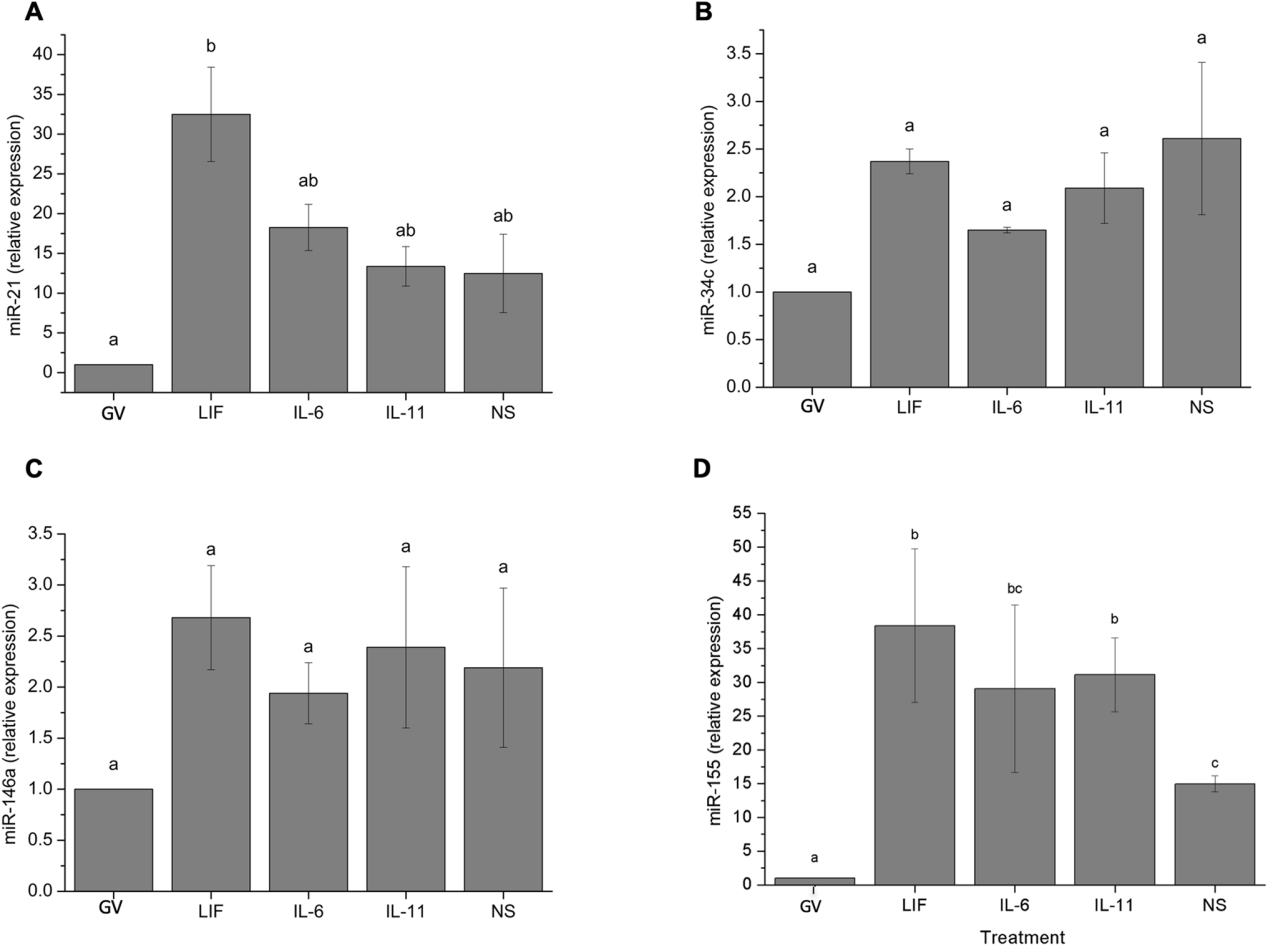
Relative expression of miR-21 (**A**), miR-34c (**B**), miR-146a (**C**), and miR-155 (**D**) transcripts in bovine cumulus cells. a–c: Different letters indicate statistically significant differences between treatments (*P* < 0.05). *GV* immature oocytes at germinal vesicle, *LIF* oocytes in vitro matured with 25 ng/mL LIF, *IL-6* oocytes in vitro matured with 10 ng/mL IL-6, *IL-11* oocytes in vitro matured with 5 ng/mL IL-11; and *NS* oocytes in vitro matured in H-TCM199 without any other supplements. Data are shown as mean ± SEM.

In order to investigate the potential mechanisms underlying the effects of IL-6 family cytokines on cellular function in the context of cumulus cells and the oocyte, we first evaluated the levels of miRNA transcripts in cumulus cells from COCs matured in the presence of LIF, IL-6, IL-11 or in non-supplemented IVM media. Results are shown in Fig. 1. Significantly higher (*P* < 0.05) miR-21 transcript levels were observed in cumulus cells from COCs in vitro matured with LIF when compared to the expression levels of miR-21 in cumulus cells from GV oocytes (Fig. 1A). However, no significant differences in miR-21 expression were observed between cumulus cells from immature oocytes and matured oocytes with no supplementation or supplemented with IL-6 or IL-11. Whereas the expression of miR-155 was significantly (*P* < 0.05) higher in cumulus cells from MII-than in those from GV-oocytes, levels of miR-34c and miR-146a did not vary when their expression was assessed in cumulus cells, regardless of the meiotic stage or IVM treatment (Fig. 1B, C and D).

**Figure 2:**
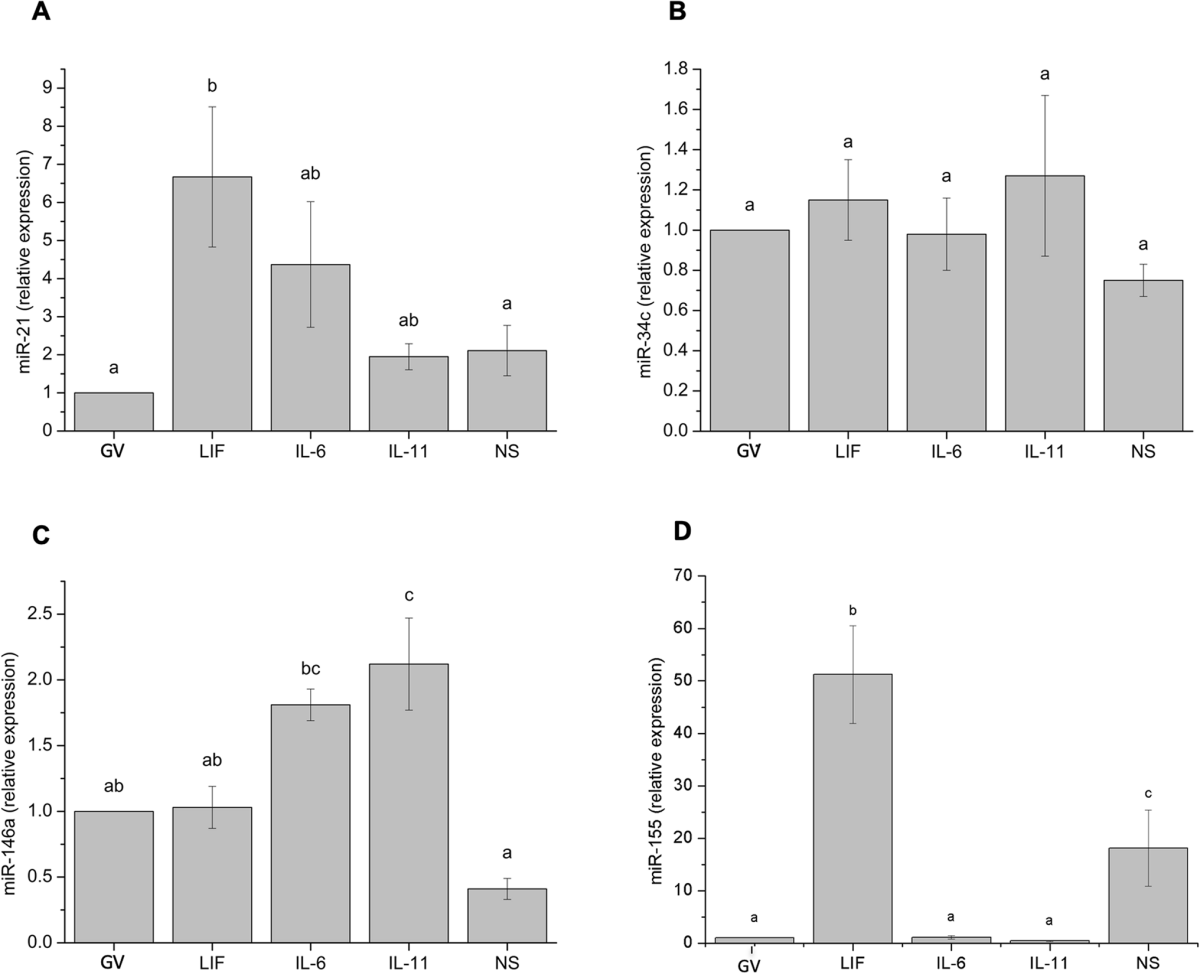
Relative expression of miR-21 (**A**), miR-34c (**B**), miR-146a (**C**), and miR-155 (**D**) transcripts in bovine in vitro matured oocytes. a–c: Different letters indicate statistically significant differences between treatments (*P* < 0.05). *GV* immature oocytes at germinal vesicle, *LIF* oocytes in vitro matured with 25 ng/mL LIF, *IL-6* oocytes in vitro matured with 10 ng/mL IL-6, *IL-11* oocytes in vitro matured with 5 ng/mL IL-11; and *NS* oocytes in vitro matured in H-TCM199 without any other supplements. Data are shown as mean ± SEM.

Levels of miRNA expression assessed in oocytes are shown in Fig. 2. Expression of miR-21 and miR-155 significantly (*P* < 0.05) increased when oocytes were in vitro matured with LIF. No differences in miR-21 or miR155 expression levels were observed between immature oocytes and oocytes in vitro matured with IL-6 or IL-11 (Fig. 2A,D). miR-146a expression was significantly (*P* < 0.05) higher when IL-11 was added to the maturation medium (Fig. 2C) than in the other treatments, but similar in oocytes matured with IL-6. Expression levels of miR-34c in oocytes were not affected by the presence of LIF, IL-6 or IL-11 (Fig. 2B).

### Effects of the addition of LIF during IVM on in vitro embryo developmental competence and levels of specific transcripts in blastocysts

LIF demonstrated the most consistent effects on the expression of different miRNAs through development. To determine whether LIF supplementation during oocyte maturation also affected development, we assessed several established parameters of developmental competence. No significant differences were observed between LIF treatments and control embryos in terms of cleavage rates, blastocyst yield at day 7 and day 8 (Table 1). However, significantly higher hatching rate was observed in those blastocysts derived from oocytes in vitro matured in presence of LIF when compared to those derived form control oocytes.

**Table 1:** Embryo development of bovine oocytes in vitro matured in a medium supplemented with 25 ng/mL of LIF.

	n	Cleavage	D7 Blastocyst	D8 Blastocyst	D8 Blastocyst			
					n_D8_	Non-expanded	Expanded	Hatched
Control	798	86.46 ± 2.33	26.00 ± 2.55	35.04 ± 3.01	284	17.22 ± 2.34	36.36 ± 4.21	46.41 ± 3.88^a^
LIF	818	84.01 ± 2.90	24.86 ± 2.48	30.72 ± 1.98	272	11.61 ± 2.65	31.33 ± 2.60	57.06 ± 2.89^b^

Values are given as percentage ± SEM.

Treatment groups: Control: bovine oocytes matured in TCM-199 supplemented with 10% FBS, 10 ng/mL EGF and 50 µg/mL gentamycin. LIF: bovine oocytes matured in TCM-199 supplemented with 10% FBS, 10 ng/mL EGF and 50 µg/mL gentamycin and 25 ng/mL of LIF.

Results of Total Cell Number (TCN), number of ICM cells, number of TE cells and apoptotic rate (AR) of Day 8 blastocysts derived from oocytes in vitro matured in the presence of 25 ng/mL of LIF are shown in Table 2. While no differences in the TCN, TE cell number and AR were observed regardless of the IVM treatment or blastocyst stage, the number of cells of the ICM was significantly higher in non-expanded, expanded and hatching/hatched blastocysts derived from oocytes matured in medium supplemented with LIF when compared to their respective control blastocyst stages. Besides, a significant increase in TCN, ICM cell number and TE cell number was observed as the blastocyst stage progessed from non-expanded to hatched, being higher at hatching/hatched stages. When apoptotic rate was assessed, no differences were observed between D8 blastocysts derived from oocytes in vitro matured with or without the presence of LIF, regardless of the developmental stage. However, AR was significantly higher in non-expanded blastocysts compared to expanded and hatching/hatched blastocysts, regardless of the maturation treatment.

**Table 2:** Analysis of TCN, nº of cells in the ICM and TE and AR of Day 8 blastocysts derived from oocytes IVM in a medium supplemented with 25 ng/mL of LIF.

	Day 8 blastocysts														
	n			TCN ± SEM			ICM cell number ± SEM			TE cell number ± SEM			AR ± SEM		
	Bl	Exp	Hd	Bl	Exp	Hd	Bl	Exp	Hd	Bl	Exp	Hd	Bl	Exp	Hd
Control (n = 47)	18	16	13	84.7 ± 2.6^a,c^	148.8 ± 5.3^a,d^	199.7 ± 9.1^a,e^	8.8 ± 0.8^a,c^	14.2 ± 0.7^a,d^	25.9 ± 2.5^a,e^	75.9 ± 2.1^a,c^	125.0 ± 3.8^a,d^	173.8 ± 8.6^a,e^	8.3 ± 0.3^a,c^	4.5 ± 0.2^a,d^	4.2 ± 0.4^a,d^
LIF (n = 62)	20	26	16	86.7 ± 3.1^a,c^	134.8 ± 4.4^a,d^	202.1 ± 6.1^a,e^	12.8 ± 1.0^b,c^	22.6 ± 1.8^b,d^	34.9 ± 2.3^b,e^	74.5 ± 2.4^a,c^	116.6 ± 4.7^a,d^	163.8 ± 6.7^a,e^	8.9 ± 0.7^a,c^	3.8 ± 0.1^a,d^	3.6 ± 0.5^a,d^

Treatment groups: Control: bovine oocytes matured in TCM-199 supplemented with 10% FBS, 10 ng/mL EGF and 50 µg/mL gentamycin. LIF: bovine oocytes matured in TCM-199 supplemented with 10% FBS, 10 ng/mL EGF and 50 µg/mL gentamycin and 25 ng/mL of LIF.

*Bl* blastocyst, *Exp* expanded blastocyst, *Hd* Hatching/Hatched blastocyst. Data are shown as mean ± SEM, *TCN* total cell number, *ICM* inner cell mass, *TE* trophectoderm, *AR* Apoptotic rate.

^a,b^Values within columns with different superscripts differ significantly (*P* < 0.05); ^c,d,e^Values for each valuable within rows with different superscripts differ significantly (*P* < 0.05).

**Figure 3:**
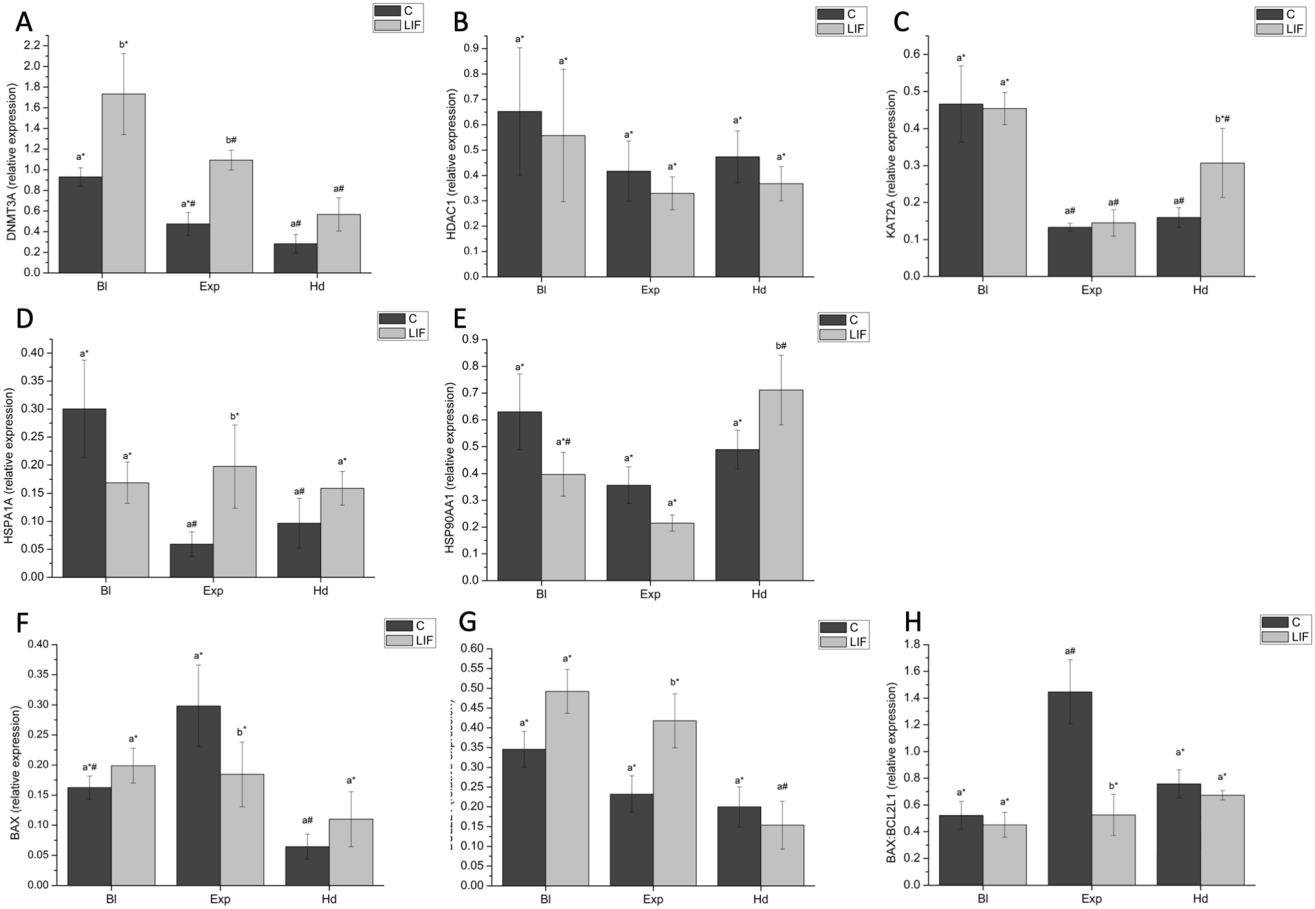
(**A**) *DNMT3A*, (**B**) *HDAC1*, (**C**) *KAT2A*, (**D**) *HSPA1A*, (**E**) *HSP90AA1*, (**F**) *BAX*, (**G**) *BCL2L1* profiles and (**H**) *BAX:BCL2L1* ratio in blastocysts derived from bovine oocytes in vitro matured with or without LIF supplementation. Different letters indicate statistically significant differences between treatments (*P* < 0.05), and different symbols indicate differences between developmental stages within a given treatment. *DNMT3A*, DNA methyltransferase 3 alpha; *KAT2A*, Lysine acetyltransferase 2A; *HDAC1*, Histone deacetylase 1; *HSPA1A*, Heat shock 70 kDa protein; *HSP90AA1*, Heat shock protein HSP 90-alpha; *BAX*, BCL2 associated X apoptosis regulator; *BCL2L1*, BCL2 like 1; Bl, Blastocyst; Exp, Expanded blastocyst; Hd, Hatching/Hatched blastocyst. Data are shown as mean ± SEM.

With regard to the effects of LIF supplementation during in vitro maturation on gene expression patterns of blastocysts, similar transcript levels were observed regardless of the LIF treatment, except for the expression levels of *DNMT3A,* which were the highest in the LIF group. Relative mRNA abundances of *DNMT3A*, *HSPA1A* and *BCL2L1* genes were significantly (*P* < 0.05) higher in expanded blastocysts arising from bovine oocytes in vitro matured with LIF. However, LIF supplementation during in vitro maturation significantly decreased (*P* < 0.05) *BAX* expression and *BAX:BCL2L1* ratio in derived D8 expanded blastocysts. Similar transcript levels of all genes analyzed were observed in hatching and hatched blastocyst except for the significantly (*P* < 0.05) higher abundance of *KAT2A* and *HSP90AA1* transcripts (Fig. 3).

### Effects of LIF supplementation during IVM on levels of specific mRNA transcripts in oocytes and early embryo stages

**Figure 4:**
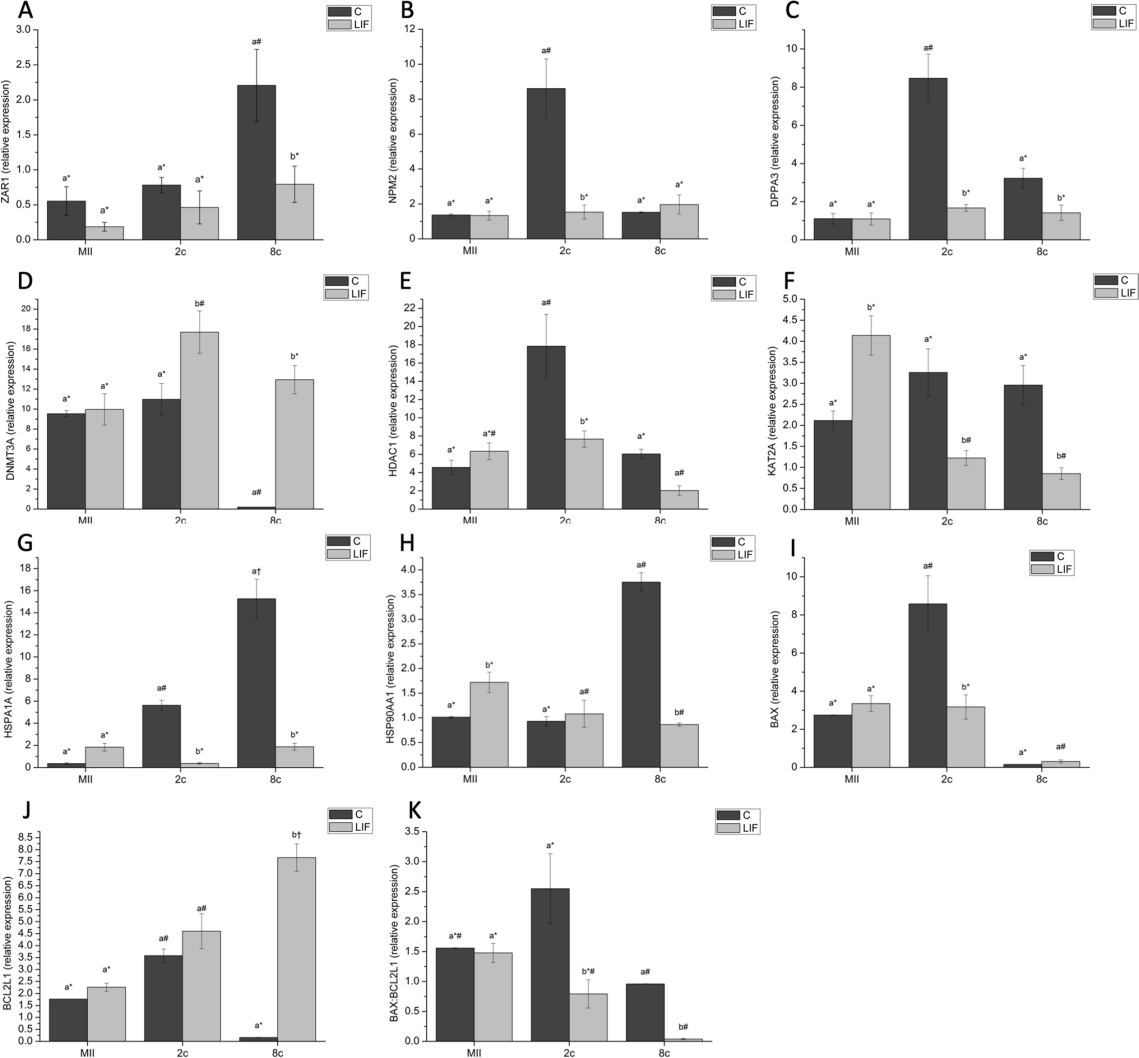
(**A**) *ZAR1*, (**B**) *NPM2* (**C**), *DPPA3* (**D**) *DNMT3A*, (**E**) *HDAC1,* (**F**) *KAT2A,* (**G**) *HSPA1A*, (**H**) *HSP90AA1,* (**I**) *BAX*, and (**J**) *BCL2L1* profiles and (**K**) *BAX:BCL2L1* ratio in MII oocytes, 2- and 8-cell embryos from control and LIF in vitro matured oocytes. Different letters indicate statistically significant differences between treatments (*P* < 0.05), and different symbols indicate differences between developmental stages within a given treatment. *ZAR1*, zygote arrest 1; *NPM2,* nucleoplasmin 2; *DPPA3,* developmental pluripotency associated 3; *DNMT3A,* DNA methyltransferase 3 alpha; *KAT2A,* Lysine acetyltransferase 2A; *HDAC1,* Histone deacetylase 1; *HSPA1A*, Heat shock 70 kDa protein; *HSP90AA1*, Heat shock protein HSP 90-alpha; *BAX*, BCL2 associated X apoptosis regulator; *BCL2L1,* BCL2 like 1; MII, metaphase II; 2c, 2-cell embryos; 8 c, 8-cell embryos. Data are shown as mean ± SEM.

In addition to miRNA expression, we determined the effects of LIF treatment during in vitro maturation on the expression of several key developmental genes implicated in different aspects of cellular function during subsequent development. Analysis of MII oocytes revealed no differences in the expression of the genes analyzed, except for the expression of *KAT2A* and *HSP90AA1* genes that were increased after LIF treatment. The relative abundance of *NPM2, DPPA3, HDAC1, KAT2A, HSPA1A* and *BAX* transcripts in 2-cell embryos obtained from oocytes matured with LIF was lower (*P* < 0.05) than those of the control group. However, the relative expression of *DNMT3A* (*P* < 0.05) was significantly higher in 2-cell embryos derived from LIF oocytes. Lower expression levels were observed for *ZAR1, DPPA3*, *KAT2A, HSPA1A* and *HSP90AA1* genes in 8-cell embryos arising from oocytes matured with LIF. In contrast, the expression of both *DNMT3A* and *BCL2L1* was significantly increased in 8-cell embryos derived from oocytes matured with LIF (Fig. 4).

## Discussion

Oocytes and their surrounding cumulus cells undergo many complex processes that require regulated changes in transcript and miRNA levels at specific stages^7,16,38^. Cytokines such as those in the IL-6 family are widely recognized to be important initators of cellular events, particularly in the follicle. In order to better understand the potential effects of this cytokine family on key miRNAs known to be expressed in COCs, we investigated the relative expression patterns of miR-21, miR-146a, miR-34c and miR-155 in bovine oocytes and their surrounding cumulus cells of COCs in vitro matured in media supplemented with LIF, IL-6 and IL-11. Results revealed that COCs treated with LIF showed higher expression of miR-21 in both cumulus cells and oocytes when compared to immature oocytes. Increased expression of miR-21 has previously been described in COCs after 24 h in culture under serum free conditions, without supplements or in culture supplemented with LIF alone^12^ and miR-21 is recognized as a highly abundant miRNA in cumulus cells^9,38^ that is present throughout folliculogenesis^38^. In addition, miR-21 is implicated in apoptosis, cell cycle arrest, tumor suppression and chromosome alignment^36^. Suppression of miR-21 is known to result in lower numbers of oocytes that complete meiosis, and is critical for pig oocyte competence^39^. miR-146a, which was increased by both LIF and IL-6, also functions as an apoptosis regulator^40,41^. In a recent study, Chen et al.^42^ reported that increased miR-146a expression promotes apoptosis of granulosa cells in patients suffering from premature ovarian failure. This suggests that IL-6 or IL-11 driven increases in miR-146a may increase cumulus apoptosis in some circumstances.

The induction of miR-155 expression in oocytes^13^ and cumulus cells^9^ has been previously described in bovine COCs matured under standard IVM conditions. An increase in miR-155 expression by inflammatory cytokines has previously been demonstrated in other cell types^43^. In our study, all gp130 cytokines tested increased miR-155 expression in cumulus cells but only LIF increased its expression in oocytes. One validated target of miR-155 that is present in COCs is inositol polyphosphate 5-phosphatase 1 (INPP5D)^44^. miR-155 mediated suppression of INPP5D would increase AKT activity^45^, and stimulate the metaphase I to metaphase II transition^46^.

The effects of cytokine treatment on the expression of potentially beneficial miRNAs observed in this study suggested that LIF has significant potential to enhance oocyte competency. To further explore this possibility, three additional experiments were conducted to evaluate the effects of LIF on embryo development and gene expression. Previous studies reported higher rates of cleavage, blastocyst formation and TCN in blastocysts in bovine oocytes that received LIF during IVM^32^. In our study, the total cleavage and blastocyst rates after LIF treatment were not different from controls, as observed by Wasielak et al.^35^ in pigs. However, in vitro maturation in a LIF supplemented medium improved the rate of hatching and the number of ICM cells at advanced stages of preimplantation embyo development, without triggering changes in total or TE cell counts, or in the incidence of apoptosis. Hatching competence has been recognized as an important parameter of embryo quality^47^ while a higher cell count in the ICM has been shown to improve the survivability of embryos after transfer to recipients^48^ since small ICM numbers in IVP bovine embryos likely contribute to many of the early pregnancy failures observed in cattle receiving these embryos^49^.

In order to further explore the potential effects of LIF on the expression of developmentally important genes, we next examined the effect of in vitro maturation with LIF on the mRNA level of MEG (*ZAR1*, *NPM2* and *DPPA3*) in bovine MII oocytes and early embryo stages. Oocytes rely on maternal factors before transcription is resumed through the process called EGA. Our results indicated that LIF suppresses the expression of *DPPA3, ZAR1* and *NPM2* in 2 cell- and/or 8 cell-embryos. Moreover, oocytes matured with LIF showed similar pattern of expression for *DPPA3, ZAR1* and *NPM2* throughout early embryo development (MII oocytes, 2- and 8-cell embryos) when oocytes were matured with LIF. Similar to our results, Wasielak et al.^35^ did not observe differences in *ZAR1*, *NPM2* and *DPPA3* mRNA profiles throughout in vitro embryo development when porcine oocytes were in vitro matured in a medium supplemented with LIF.

DPPA3 protein regulates the maintenance of differently methylated regions in the germline^50^. Transcripts for this protein are highly abundant in the oocyte, with decreasing levels observed during EGA^51^. By maintaining *DPPA3* steady state mRNA levels in bovine oocytes and in vitro-derived 2- and 8-cell embryos, LIF may help regulate levels of DNA methylation. ZAR1 protein participates in chromatin-dependent transcriptional regulation during the maternal-to-zygotic transition^52^ while *NPM2* participates in nucleolar and nuclear organization^53^. In cattle, *ZAR1* and *NPM2* transcripts are present in immature and mature oocytes and their mRNA expression decreases form zygote to the 8-cell stage^54,55^ because of EGA during the transition from 8-cells to 16-cells. Increased expression of *ZAR1* and *NPM2* observed in embryos derived from control IVM oocytes may be due to incomplete EGA in some embryos. However, the presence of LIF during IVM significantly decreased *ZAR1* and *NPM2* mRNA expression at the 8 cell-stage, which suggests potential roles in ameliorating incomplete EGA in these embryos.

*DNMT3A*, another important gene during EGA^56,57^ was significantly increased in 2- and 8-cell embryos, blastocysts and expanded blastocysts derived from in vitro oocytes matured with LIF. In contrast, no changes in *HDAC1* mRNA expression were observed throughout embryo development except for 2-cell embryos, in which LIF suppressed *HDAC1* expression. The increased *DNMT3A* levels found in our study suggest the potential for higher methylation activity in embryos derived from LIF treated oocytes. LIF stimulates the Janus kinase/signal transducer and activator of transcription 3 (JAK/STAT3) pathway which in turn drives expression of *DNMT3A, DNMT3B, DNMT3L* and suppresses that of histone deacetylases (*HDACs*) during mouse somatic cell reprogramming^58^. *DNMT3* family members are implicated in maternal imprinting^59,60^, and suppression of *DNMT3A* levels is associated with the loss of methylation on maternally imprinted genes and is associated with embryonic death. Our study suggests that LIF may help drive mechanisms through DNMT3 expression that favour the establishment of appropriate DNA methylation patterns in this crucial window. The variable expression observed for lysine acetyltransferase 2A (*KAT2A*) in the LIF-treated group is consistent with previous studies of *KAT2A* through embryo development in bovine^61^ and mouse^62^.

Since apoptosis is another important process in the developing embryo that may be regulated by LIF and by some of the miRNAs studied, we next examined the ratio of *BAX:BCL2L1* transcripts, two important participants in apoptosis, as one indicator of the cellular effects of LIF in this context^63^. LIF has been previously described to exert an anti-apoptotic effect by downregulating *BAX*, either via STAT3^64^ or PI3K/AKT pathways^65^. Examined individually, significantly lower *BAX* expression levels were observed in 2 cell-embryos derived from control and LIF oocytes, whereas LIF treated oocytes increased *BCL2L1* expression in 8 cell-embryos and expanded blastocysts. Yang and Rajamahendran^66^ have reported that the expression of *BCL2L1* is higher than that of *BAX* in good quality embryos, whereas the opposite occurs in their low quality counterparts. In our study, *BAX:BCL2L1* ratios indicated that 2-cell and 8-cell embryos and expanded blastocysts obtained from oocytes treated with LIF had higher expression levels of *BCL2L1* than of *BAX* (*P* < 0.05). However, the apoptosis detected by TUNEL analysis in expanded blastocyts was likely independent of the expression of *BCL2L1* or *BAX* genes, as observed previously^67^.

Other pathways that contribute to improved embryo survival include the expression of heat shock proteins^68^. Heat shock protein 70 kDa (*HSPA1A*) is spontaneously transcribed during EGA^69^ and, during early embryo development, HSPA1A appears to be the first system of protection and survival through anti-apoptotic effects^70^. Steady-state levels of *HSPA1A*-mRNA were higher for 2-cell embryos than for blastocysts as previously reported for *HSPA1A*^71^. This likely reflects large-scale turnover of maternally derived mRNA as embryos advance through development^72^. However, in vitro maturation with LIF increased *HSPA1A* expression in expanded blastocysts, possibly through activation of PI3K-AKT pathway by LIF and increased *HSPA1A* transcription^73^. We postulate that LIF may help suppress apoptosis through this pathway. Similar patterns of expression were observed for gene *HSP90AA1,* but increases were only significant for MII oocytes and hatched blastocysts from oocytes matured with LIF.

In conclusion, our results demonstrate that LIF contributes to improved in vitro embryo development by actively modulating the expression of several key miRNAs and genes that we have characterized here particularly in the period just prior to EGA. While these changes did not have significant effects on some parameters such as blastocyst rate, blastocysts obtained after 8 days of in vitro culture showed a higher hatching abilility, together with a higher number of ICM cells. It would therefore be reasonable to postulate that in vitro maturation in the presence of LIF may optimize the synthesis and storage of important maternal transcripts and miRNAs during oocyte maturation or after fertilization leading to improved oocyte quality. While the exact mechanisms through which LIF exerts its effects during oocyte maturation have yet to be elucidated, the results reported herein support further research in this area in efforts to identify the appropriate combinations of endogenous signaling molecules necessary to optimize subsequent development and improve reproductive efficiency.

## Materials and methods

### Chemicals and suppliers

All chemicals and reagents were purchased from Sigma Chemical Co. (St. Louis, Mo, USA) unless otherwise stated.

### Oocyte collection and in vitro maturation

Cow ovaries were transported from a local slaughterhouse in phosphate-buffered saline (PBS) at 35–37 °C. Cumulus-oocyte complexes (COCs) were aspirated and collected in serum-free, 1 M HEPES-buffered TCM-199 (H-TCM199) collection medium supplemented with 2 IU/mL Hepalene (LEO Pharma, ON, Canada), 1 μg/mL gentamycin and 0.1% polyvinyl alcohol (PVA). COCs were washed twice in H-TCM199 maturation medium supplemented with 22 μg/mL sodium pyruvate, 10 μg/mL gentamycin and 0.1% PVA. Groups of up to 20 COCs were placed in 100 µL drops of maturation medium covered with mineral oil, and cultured in a humidified atmosphere at 38.5 °C and 5% CO_2_. After 24 h, cumulus cells were removed from the oocytes of each treatment mechanically in PBS with 0.1% PVA and pelleted by centrifugation at 600 × *g* and room temperature for 6 min. Cumulus free oocytes were treated with 2 mg/mL hyaluronidase to remove any remaining cumulus cells, and washed in PBS-PVA. Cumulus free oocytes and the corresponding cumulus cells of each treatment were immediately snap frozen in liquid nitrogen and stored at − 80 °C.

The in vitro protocols followed for the expression of specific mRNAs and for evaluation of developmental outcomes have been described elsewhere^74^. Briefly, COCs were obtained by aspirating 3–10 mm follicles. Only COCs with three or more layers of cumulus cells and a homogeneous cytoplasm were selected for in vitro maturation. After three washes in modified Dulbecco’s PBS (PBS supplemented with 0.036 mg/mL pyruvate, 0.05 mg/mL gentamycin and 0.5 mg/mL bovine serum albumin (BSA), groups of up to 20 COCs were placed in 100 µL drops of maturation medium and cultured for 24 h at 38.5 °C in a 5% CO_2_ humidified air atmosphere. The maturation medium consisted of TCM-199 supplemented with 10% (v/v) fetal bovine serum (FBS), 10 ng/mL epidermal growth factor (EGF) and 50 µg/mL gentamycin.

### In vitro fertilization and embryo culture

In vitro matured oocytes were in vitro fertilized and cultured as described elsewhere^75^. Briefly, frozen-thawed spermatozoa from Asturian bulls (ASEAVA, Llanera, Asturias, Spain) of proven fertility were used in all the experimental procedures. High motility and good morphology spermatozoa were obtained by centrifuging frozen/thawed sperm at 300 × *g* and room temperature for 10 min on a discontinuous gradient composed by 1 mL of 40% and 1 mL of 80% BoviPure (Nidacon Laboratories AB, Göteborg, Sweden) according to the manufacturer’s instructions. Viable spermatozoa collected from the bottom were washed with 3 mL of BoviWash (Nidacon International, Göteborg, Sweden) and pelleted by centrifugation at 300 × *g* for 5 min. Spermatozoa were counted in a Neubauer chamber and diluted in an appropriate volume of fertilization medium (Tyrode’s medium supplemented with 25 mM bicarbonate, 22 mM Na-lactate, 1 mM Na-pyruvate, 6 mg/mL fatty acid-free BSA and 1 mg/mL heparin-sodium salt) to a final concentration of 1 × 10^6^ spermatozoa/mL. One hundred μL droplets of diluted sperm were prepared under mineral oil and were co-incubated with 20 oocytes/droplet at 38.5 °C, 5% CO_2_ and high humidity.

After 18–20 h, presumptive zygotes were stripped of remaining cumulus cells by pipetting and cultured in groups of 20 in 20 µL drops of IVC medium, consisting of Synthetic Oviduct Fluid (Caisson Labs, Smithfield, USA) supplemented with 0.96 μg/mL BSA, 88.6 μg/mL Na-pyruvate, 2% non-essential amino acids, 1% essential amino acids, 0.5% gentamycin and 2% FBS under mineral oil. Presumptive zygotes were incubated at 38 °C in a humidified 5% CO_2_ and 5% O_2_ atmosphere for 8 days. Embryo development was recorded on day 2 (cleavage), and days 7 and 8 (blastocysts) post-insemination (pi). Day 8 embryos were classified in terms of the degree of blastocoel expansion into three groups according to the IETS standards: (a) early blastocysts (stage code 5) and blastocysts (stage code 6); (b) expanded blastocysts (stage code 7); and (c) hatching (stage code 8) or hatched blastocysts (stage code 9).

### RNA extraction, reverse transcription and quantitative real-time PCR analysis for miRNA

Protocols used for RNA extraction, reverse transcription and quantitative real-time PCR analysis for miRNA have been decribed elsewhere^12^. Total RNA, including small RNA, was isolated using the miRNeasy Micro kit (Qiagen; Mississauga, ON, USA) according to the manufacturer’s protocol. RNA was isolated from pools of 30 COCs as cumulus or oocyte fractions for use with miRNeasy kit. miRNA was extended by polyadenylation and reverse transcribed using the qScript microRNA cDNA Synthesis Kit (Quantabio). RT-qPCR was performed with a CFX96 Touch Real-Time PCR Detection System (BioRad Laboratories, Inc., Hercules, CA) using PerfeCTa SYBR Green SuperMix (Quantabio). cDNAs encoding miRNAs were amplified with a gene-specific forward primer (Table 3) and PerfeCTa Universal PCR Primer (Quantabio). Two µL of cDNA template were used for each reaction. Efficiencies were calculated by standard curve for all primers designed in this study and gene expression was calculated by the efficiency-corrected ΔΔCt method^76^. miRNAs were normalized to snRNA U6, which has been previously shown to be suitable for oocytes^16^ and is stably expressed in cumulus cells. In each run, there were three technical replicates from each of three biological replicates per individual gene. Further, negative controls for the template and for the reverse transcription were also included and amplified by PCR to ensure no cross-contamination. Expression of each miRNA is shown as relative to the abundance of that miRNA in the immature oocyte group.

**Table 3:** Specific primer sequences used in this study (miRNA).

Primer name	Primer sequence (5′–3′)	Length (bp)
bta-miR-21	GCTAGCTTATCAGACTGATGTTGACTAAA	29
bta-miR-146a-5p F	TGAGAACTGAATTCCATAGGTTG	23
GMS hsa-miR-155-5p	TGCTAATCGTGATAGGGGTAAA	22
bta-miR-34c-5p	AATCACTAACCACACGGCCAGG	22
RNU6	CGCAAGGATGACACGCAAATTCGTGAAGCGTTCCATATTTTT	42

### RNA extraction, reverse transcription and quantitative real-time PCR analysis for mRNA

Protocols used for RNA extraction and reverse transcription have been decribed elsewhere^75^. GV and MII oocytes were pooled in groups of 20 and embryos in groups of 5. Samples were washed three times with Dulbecco’s PBS supplemented with 0.01% PVA at 38.5 °C and then pipetted into 1.5 mL microtubes. Immediately, tubes were plunged into liquid nitrogen and stored at − 80 °C until further processed.

First, poly-(A)-RNA was extracted using Dynabeads mRNA Direct Extraction Kit (Invitrogen, Oslo, Norway), following the manufacturer’s instructions with minor modifications^77^. For poly-(A)-RNA extraction, pooled samples were lysed in 50 μL of lysis buffer at room temperature for 5 min with gently pipetting, and the fluid lysate was then hybridized with 10 μL of pre-washed beads for 5 min with gently shaking. After hybridization, poly-(A)-RNA-bead complexes were washed twice in 50 μL of Washing Buffer A and two more times in 50 μL of Washing Buffer B. Next, samples were eluted in 16 μL of Elution Buffer (Tris HCl) and heated to 70 °C for 5 min. Immediately after extraction, quantity and purity of extracted RNA was determined by spectrophotometry at 260 nm and 280 nm (Epoch Microplate Spectrophotometer, BioTek; Winooski, VT, USA). For Reverse Transcription (RT), 10 ng of extracted mRNA (Abs_260_/Abs_280_ ≥ 1.7) were mixed with 4 μL of qScript cDNAsupermix (Quanta Biosciences; Gaithersburg, MD, USA) containing oligo-dT primers, random primers, dNTPs and qScript reverse transcriptase. The RT reaction was run for 5 min at 25 °C, followed by 1 h at 42 °C to allow the RT-PCR of mRNA and 10 min at 70 °C to denature the reverse transcriptase enzyme. After RT, the resultant cDNA was diluted in 25 μL of Tris HCl (elution solution).

Quantification of relative abundance of mRNA transcripts was performed by the qPCR method described elsewhere^75^ using a 7500 Real Time PCR System (Applied Biosystems, Foster City, California, USA). The qPCR reaction mix contained 10 μL of Fast SYBR Green Master Mix (Applied Biosystems, Foster City, California, USA), 2 μL of each primer (500 nM; Life Techno Life Technologies, Madrid, Spain) and 2 μL of cDNA template. Nuclease-free water was added to a final volume of 20 μL. PCR amplification consisted of one cycle of denaturation at 95 °C for 10 min, 45 cycles of amplification with a denaturation step at 95 °C for 15 s, an annealing step at 60 °C (the appropriate annealing temperature of primers) for 1 min, and a final extension step at 72 °C for 40 s. Fluorescence data were acquired during the final extension step. To verify the identity of the amplified PCR product, melting curve analysis and gel electrophoresis in a 2% agarose gel containing 0.6 μg/mL ethidium bromide) were performed. The melting protocol consisted of heating the samples from 50 to 95 °C and holding at each temperature for 5 s while monitoring the fluorescence. In each run, three technical replicates per sample and individual gene were included. Further, negative controls for the template and for the reverse transcription were also included and amplified by qPCR to ensure that no cross-contamination occurred.

The comparative threshold cycle (Ct) method was used to quantify the relative expression of ten candidate genes (*ZAR1*; *NPM2*; *DPPA3; DNMT3A*; *KAT2A*; *HDAC1*; *HSPA1A*; *HSP90AA; BAX*; *BCL2L1*) for GV, MII, 2-cell and 8-cell embryos and seven genes (*DNMT3A*; *KAT2A*; *HDAC1*; *HSPA1A*; *HSP90AA1; BAX*; *BCL2L1*) for blastocysts, normalized to the endogenous control housekeeping (HK) genes: glyceraldehyde-3-phosphate dehydrogenase (*GAPDH*) and H2A histone family member Z (*H2AFZ*). The threshold cycle, which is set in the log-linear phase, reflects the PCR cycle number at which the fluorescence generated within a given reaction is just above background fluorescence. Within this region of the amplification curve, a difference of one cycle is equivalent to doubling of the amplified PCR product. According to the comparative Ct method, the ΔCt value was determined by subtracting the mean between *GAPDH* and *H2AFZ* Ct values for each sample from the Ct value of each target gene of the sample for each replicate separately. Fold differences in relative transcript abundance were calculated for target genes assuming an amplification efficiency of 100% and using the formula 2^−(ΔΔCt)^^78^. Calculation of ΔΔCt involved the subtraction of the ΔCt value of untreated GV (in experiment 2) and early blastocysts (in experiment 3) from all the other ΔCt sample values. Primer sequences (Life Technologies, Madrid, Spain), amplicon sizes and GenBank accession numbers for each gene are provided in Table 4. Non-template controls were not amplified or returned a Ct value 10 points higher than the average Ct value for each genes. The experiment was repeated independently three times.

**Table 4:** Primers used for reverse transcription–quantitative polymerase chain reaction (gene expression).

Symbol	NCBI Gene name	Gene Bank Accession number	Primer sequences (5′–3′)	Amplicon size (bp)
*BAX*	BCL2 associated X, apoptosis regulator	NM_173894.1	F: GAGAGGTCTTTTTCCGAGTGGC	237
			R: TGTCCCAAAGTAGGAGAGGAG	
*BCL2L1*	BCL2 like 1	BC147863.1	F: CCACTTAGGACCCACTTCTGAC	188
			R: GGGTGCTTCCTACAGCTACAGT	
*DNMT3A*	DNA methyltransferase 3 alpha	NM_001206502.1	F: CCTCAGCTCCCCCTACTTATTC	199
			R: AGCTGTGAGCTTACTCCTGAGC	
*DPPA3*	Developmental pluripotency associated 3	NM_001111108.2	F: TGGCTACTCTTCATCCCCTACA	230
			R: TCTAGGGTCCAGGTTGGGTT	
*GADPH*	Glyceraldehyde-3-phosphate dehydrogenase	NM_001034034.2	F: AGTCCACTGGGGTCTTCACTAC	243
			R: CAGTGGTCATAAGTCCCTCCAC	
*HDAC1*	Histone deacetylase 1	NM_001037444.2	F: CTGAGGAGATGACCAAGTACC	167
			R: CCACCAGTAGACAGCTGACAGA	
*HSPA1A*	Heat shock protein family A (Hsp70) member 1A	NM_203322.3	F: GCAGGTGTGTAACCCCATCA	181
			R: CAGGGCAAGACCAAAGTCCA	
*HSP90AA1*	Heat shock protein 90 alpha family class A member 1	NM_001012670.2	F: GTGGAGACTTTCGCCTTCCA	223
			R: TGGTGAGGGTTCGATCTTGC	
*H2AFZ*	H2A histone family, member Z	NM_174809.2	F: GCGTATTACCCCTCGTCACTTG	227
			R: GTCCACTGGAATCACCAACACTG	
*KAT2A*	Lysine acetyltransferase 2A	XM_015468132.1	F: AGGATGTGGCTACCTACAAGG	190
			R: GCACCAGCTTGTCCTTCTCTAC	
*NPM2*	Nucleophosmin/nucleoplasmin 2	NM_001168706.1	F: GGACCTGTGTTCCTCTGTGG	153
			R: CTTCACTTGTTTGACGGGCG	
*ZAR1*	Zygote arrest 1	NM_001076203.1	F: GGGAGATGCAAAGGCAAACG	216
			R: CCAAACAACAGCCTTCCACG	

### Differential staining of blastocysts

**Figure 5:**
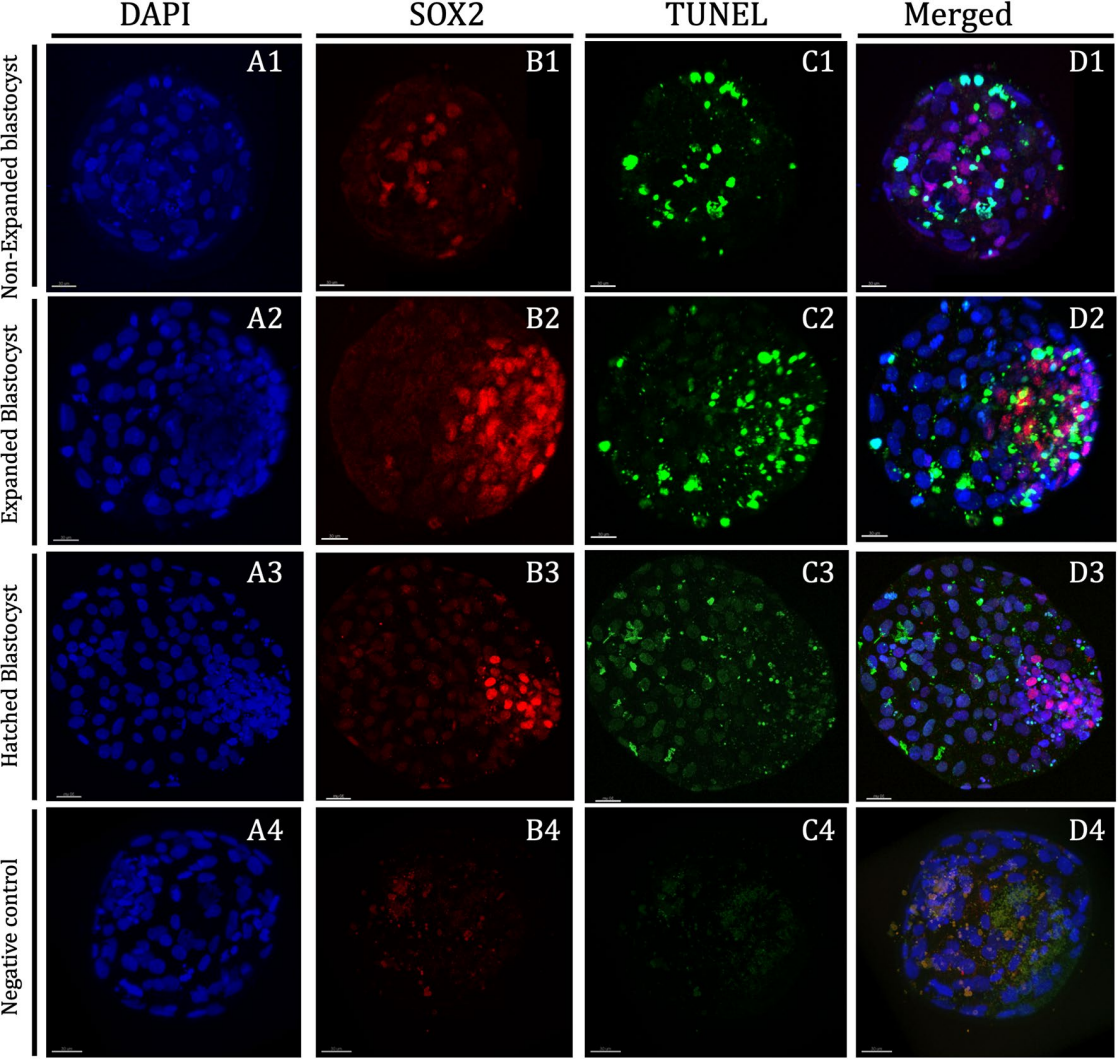
Representative pictures of D8 non-expanded, expanded and hatched blastocysts derived from oocytes in vitro-matured in the presence of 25 ng/mL of LIF. Nuclei counterstained with DAPI are displayed in blue (**A1**–**A3**), ICM is displayed in red (**B1**–**B3**) and TUNEL-positive cells are displayed in green (**C1**–**C3**). An overlay is given in (**D1**), (**D2**) and (**D3**). Non-expanded blastocyst: (**A1**), (**B1**), (**C1**), and (**D1**); expanded blastocyst: (**A2**), (**B2**), (**C2**) and (**D2**); and Hatched blastocyst: (**A3**), (**B3**), (**C3**) and (**D3**). Negative controls for SOX2 (**B4**) and TUNEL (**C4**) with nuclei counterstained with DAPI (**A4**). Negative controls comprise the omission of the primary antibody (mouse anti-SOX2 primary antibody) or no exposure to the terminal TdT enzyme (TUNEL). Scale bar: 30 μm. DAPI (406-diamidino-2-phenylindole), TUNEL (Terminal deoxynucleotidyl transferase dUTP nick end labelling).

Day 8 bovine blastocysts underwent double staining combined with TUNEL analysis as previously described by Ascari et al.^79^ with some modifications; all steps were done at 38.5 °C unless otherwise stated. Blastocysts were fixed in 2% (v/v) paraformaldehyde in PBS for 15 min at room temperature (RT). After fixation, embryos were washed at least three times in PBS and permeabilised in 0.01% Triton X-100 in PBS supplemented with 5% Normal Donkey Serum (PBS-NDS) for 1 h at RT. The embryos were washed in PBS (3×) and incubated at 4 °C overnight with mouse anti-SOX2 primary antibody (1:100; Invitrogen, CA, USA) in a humidified chamber. Afterward, the embryos were washed in PBS (3×) for 20 min and permeabilised again with 0.005% Triton X-100 in PBS-NDS for 20 min. The embryos were then incubated with the secondary antibody goat anti-mouse IgG Alexa Fluor 568 (1:500; ThermoFisher, Ma, USA) diluted for 1 h in a humidified chamber. Afterwards, the embryos were transferred to PBS-NDS-0.005% Triton X-100 for 20 min, washed in PBS (3×) and incubated in the TUNEL reaction mixture dilution following manufacturer’s instructions (in situ Cell Death Detection Kit, Fluorescein) for 1 h in the dark. Positive and negative control samples were included in each assay. Blastocysts exposed to DNase I for 15 min at RT served as positive controls and blastocysts not exposed to the terminal TdT enzyme served as negative controls. Embryos were then washed thoroughly in 0.005% Triton X-100 in PBS-NDS for 5 min, mounted on poly-l-lysine treated coverslips fitted with a self-adhesive reinforcement ring in a 3-µL drop of Vectashield containing 125 ng mL^−1^ 4′,6-diamidino-2-phenylindole (DAPI) (Vectorlabs, Burlingame, CA), and flattened with a slide. The preparation was sealed with nail varnish and stored at 4 °C protected from light until observation within the following up to 3 days. Confocal images in serial sections separated by 0.5 µm were captured with a confocal laser scanning microscope (Leica TCS SP5, Leica Microsystems CMS GmbH, Mannheim, Germany) to examine ICM cell’s nucleus (SOX2-Alexa Fluor 568; excitation 561 nm), cell nucleus (DAPI; excitation 405 nm) and DNA fragmentation (fluorescein isothiocyanate-conjugated TUNEL label; excitation 488 nm). TCN, ICM cell number, and apoptotic cells were analysed using the Imaris 9.2 software (Oxford Instruments, UK). Individual nuclei were counted as assessed as intact (TUNEL(−); blue/red stain) or fragmented (TUNEL(+), green stain) DNA, TE cells (SOX2(−); blue stain) or ICM cells (SOX2(+); red stain) (Fig. 5). The AR was calculated as the ratio of TUNEL(+) cells/total number of cells.

### Experimental design

#### Effect of IL-6, IL-11 and LIF on miRNA expression on oocytes and cumulus cells

miRNA expression was evaluated in oocytes and cumulus cells from COCs after 24 h in culture under serum free conditions, without hormones, and compared to cultures supplemented with LIF alone, IL-6 alone or IL-11 alone in order to eliminate any effect of the supplementation with FBS or EGF during maturation. GV oocytes served as immature oocytes. LIF (10 μg/mL, L5283-10UG) was diluted in H-TCM199 to a final concentration of 25 ng/mL. Five μg of human IL-6 recombinant protein (PHC0064; Gibco, Frederick, MD, USA) were dissolved in 100 mM acetic acid to produce a 5 μg/mL stock solution and stored at -20 °C. Aliquots of this stock solution were diluted to 10 ng/mL with H-TCM199. Two μg of human IL-11 recombinant protein (ab9553; Abcam, Cambridge, MA, USA) were reconstituted with 20 µL of sterile water to a concentration of 0.1 mg/mL and stored at − 20 °C. Aliquots of this stock solution were diluted to 5 ng/mL with H-TCM199. Upon oocyte collection, COCs were randomly assigned to five treatment groups: (1) GV, immature oocytes at germinal vesicle stage; (2) LIF, COCs matured in H-TCM199 supplemented with 25 ng/mL recombinant human LIF^32^; (3) IL-6, COCs matured in H-TCM199 supplemented with 10 ng/mL recombinant human IL-6^80^; (4) IL-11, COCs matured in H-TCM199 supplemented with 5 ng/mL recombinant human IL-11^81^; and (5) NS, Non-supplemented; COCs matured in H-TCM199. Upon collection, GV oocytes were denuded mechanically from cumulus cells and the two fractions were stored separately at − 80 °C. Post-IVM, cumulus cells were separated from the oocytes of each treatment mechanically, and only those oocytes presenting extrusion of the first polar body were considered as mature. Both fractions (matured oocytes and cumulus) were stored separately at − 80 °C until qPCR analysis (three replicates).

#### Effects of the LIF addition during IVM on in vitro embryo developmental competence and levels of specific transcripts in blastocysts

Based on the observed effects on miRNA expression in the initial studies, we next evaluated the effect of LIF treatment during maturation on subsequent embryo development. Upon oocyte collection, immature COCs were randomly allocated into two experimental groups: (1) Control, oocytes were in vitro matured as previously described; and (2) LIF, oocytes were in vitro matured using the same IVM medium supplemented with human recombinant LIF (25 ng/mL). After 24 h of IVM, oocytes were in vitro fertilized and cultured. Embryo development was recorded on day 2 (cleavage), and days 7 and 8 (blastocysts) post-insemination (pi). Day 8 embryo development was classified in terms of the degree of blastocoel expansion into three groups according to the IETS standards: (a) Non-expanded: early blastocysts and blastocysts (stage code 5 and 6); (b) Expanded: expanded blastocysts (stage code 7); and (c) Hatched: hatching or hatched blastocysts (stage code 8–9) (nine replicates). Blastocysts (stage code 6), expanded blastocysts (stage code 7), and hatched blastocysts (stage code 8–9) were fixed and immunostained to assess TCN, ICM cell number, TE cell number and AR (4 replicates). Early blastocysts (stage code 5), blastocysts (stage code 6), expanded blastocysts (stage code 7), and hatched blastocysts (stage code 8–9) were pooled in groups of 5, snap frozen in liquid nitrogen and stored at − 80 °C until RNA extraction and RT-qPCR (three replicates).

#### Effects of the LIF addition during IVM on levels of specific transcripts in oocytes and early embryo stages

LIF treatment could potentially affect the expression of multiple genes relevant to development. We therefore evaluated the expression of key maternal- and apoptosis-related genes after maturation in the presence of LIF. Upon oocyte collection, immature COCs were randomly allocated into three experimental groups: (a) GV, immature oocytes at germinal vesicle stage; (b) Control, oocytes were in vitro matured as previously described; and (c) LIF, oocytes were in vitro matured using the same IVM medium supplemented with human recombinant LIF (25 ng/mL). Upon collection, GV oocytes were denuded mechanically from cumulus cells.At 24 h of IVM, a pool of oocytes from each group were denuded from cumulus cells by gentle pipeting and 20 oocytes presenting extrusion of the first polar body were collected from control and LIF groups. The remaining oocytes were in vitro fertilized and cultured. Two-cell and 8-cell embryos were harvested from the culture medium at 31–33 hpi and 52–54 hpi, respectively. All oocyte groups as well as 2-cell and 8-cell embryos were snap frozen in liquid nitrogen and stored at − 80 °C until subsequent qRT-PCR analysis (three replicates).

### Statistical analysis

Statistical tests were performed using the statistical package IBM SPSS Version 25.0 for Windows (IBM Corp.; Armonk, NY, USA). For miRNA experiments, gene expression data collected from RT-qPCR were previously log_2_ transformed and then checked for normality using the Shapiro–Wilk's test and for homogeneity of variances using the Levene test. Differences between groups were analyzed by one-way analysis of variance (ANOVA) followed by the post-hoc Sidak’s test. For the mRNA and developmental experiments, data were also checked for normality and homogeneity of variances, as stated above. When required, data were transformed through √x or arcsin √x, prior to running two-way analysis of variance ANOVA (factors: stage and treatment) followed by post-hoc Sidak test for pair-wise comparisons. Variables were cleavage rates, blastocyst yields, TCN, ICM and TE cell count, apoptosis indexand relative transcript abundances. Data are expressed as means ± standard error of the mean (SEM). The level of significance was set at *P* ≤ 0.05.
